# The Phenomena of Digestion and Their Significance

**Published:** 1903-03-14

**Authors:** 


### Hospital Clinics and Medical Progress.

### THE PHENOMENA OF DIGESTION AND THEIR SIGNIFICANCE.

The brilliant experimental work of Pawlow and his pupils has brought to light many important facts concerned with digestion. It has also provided a physiological explanation as to the meaning of a large number of clinical observations. The importance of these discoveries was soon recognised, resulting in the award of the Nobel prize of 1901 to Professor Pawlow. His investigations go to prove that it is not the latent need of the organism for food that is the immediate cause of appetite. Real appetite sets in with eating. It is in part due to an appreciation of the taste of food. By sham feeding experiments on dogs, where the food passed down the gullet but never reached the stomach, it was shown that the conscious desire for food was awakened by the swallowing of tasty morsels. This conscious desire stimulates the digestive glands, notably the stomach and pancreas, though no food reaches the stomach; a physiological explanation of the empirical fact that the first few mouthfuls of a meal stimulate the appetite for more food.

In animals the craving for food is, when awakened, an impulse to seek it. In man it gives the appetite, sometimes latent, necessary for the enjoyment of the more solid portions of the meal. Further it has been proved by Pawlow that appetite thus aroused is the strongest exciter of the several gastric glands; for even in starving animals food introduced direct into the stomach, without attracting the animal's attention, or awakening the desire for food by sight or smell, totally fails to stimulate the gastric glands or to provoke any secretion of the digestive juices. From this fact the immense importance of the "appetite" digestive secretion is apparent. It has been called the "igniting juice" by Pawlow, for without it the food remains in the stomach for a considerable period entirely unaltered. It may be more than an hour before any gastric secretion takes place, and then only when the food contains some chemical constituent that acts directly as an exciter of gastric secretion. This explains why food eaten without appetite is a cause of gastric disturbances.

Food should therefore be eaten with interest and enjoyment. The busy man must put aside his cares and troubles when he comes to his meals. The formalities of a set meal, and the ritual observed in the partaking of the various courses of a dinner, have their importance as appealing to the psychological factor in the secretion of the digestive juices.

Extra inducements, to the proper working of the digestive glands, are the stimuli derived from the organs of taste. They play a like part in starting the initial flow of the gastric and pancreatic secretions.

Bitters, and particularly alkaline bitters, have been held to promote digestion, but want of any physiological proof of their potency has led to an unwarranted neglect of their use as a therapeutic measure. Pawlow claims that they stimulate the digestion by the unconscious comparison of their disagreeable taste with the more pleasant taste of food; that they act by awakening the appetite for food. He, therefore, regards them as useful adjuncts in securing a proper flow of the initial or "igniting" digestive juice.

Habits and tastes must also be considered in drawing up a diet scheme or prescribing any fixed regimen. Neglect of this important precaution renders impossible the proper treatment of digestive disorders. Hence, in children particularly, habits and tastes as regards food require careful education, otherwise the limits of useful food, capable of exciting a proper appetite, become so narrow that a proper variation in diet is difficult to attain.

A restriction of the amount of food taken at any one meal is important as awakening a further appetite, and hence a more copious flow of digestive juices. Pawlow shows that food given in small portions, at frequent intervals, is more potent in promoting secretory activity in the digestive glands, than when the *same* amount of food is given in one meal. Here is an explanation of the clinical value of small quantities of nourishment frequently repeated, compared with the same quantity of food given in larger amounts at longer intervals of time.

The development of appetite or zest for food enters largely into the sequence of foods chosen for the chief meal of the day. Experimental evidence shows that this sequence, first observed as an empirical custom, has a definite physiological basis of fact, and is, indeed, a rational procedure.

The meal is begun with foods designed more to promote appetite than afford nutrition. The soups that follow act directly upon the secreting mechanism of the gastric glands. Pawlow finds that among the chief chemical excitants of gastric secretion are the extractives of meat, and it is these extractives that form the principal constituents of bouillon. They carry on the stimulation of the gastric glands initiated by the psychological stimuli of appetite and taste.

The acid wines, often consumed as an accompaniment of food, provide a part of the acid necessary for the activity of the gastric ferment. The acid also plays another important part. Pawlow finds that its action upon the mucous membrane of the duodenum is a direct excitant of pancreatic secretion. Bayliss and Starling have recently shown that this is due to the acid acting on a chemical compound present in the cells of the duodenal mucous membrane. The acid sets free this compound, which then circulates in the blood until the pancreas is reached. Arrived at the pancreas this compound, termed by them "secretin," is a direct chemical excitant of pancreatic secretion. Any acid, and more particularly the hydrochloric acid of gastric juice, is thus a direct and immediate cause of pancreatic activity. Gastric digestion is the proper precursor of pancreatic digestion.

Digestion, having been started by the action of the "igniting juice" on the food arriving in the stomach, proceeds by the absorption of the products of gastric digestion. They in turn stimulate the secretory mechanism of the stomach and pancreas, and the bile-forming mechanism of the liver. The early digestion of the food by the "igniting juice" thus gains additional importance, for the products of this early digestion are exciters of digestive activity; and it is this further digestive activity that deals with the more solid courses of the meal.

The varied foods that go to make up the repast, each play a part in modifying the amount and nature of the secretion poured out from the different glands. An equilibrium however is soon established among the constituents of the digestive juices, depending on the character and chemical composition of the food injested. But the chemical agencies of digestion cannot be regarded separately. They form an alliance of a complicated nature, each secretion in turn relieving the work and supporting the activity of another. The various possibilities of digestion must therefore be taken as a whole, and clinically this should be borne in mind when weighing the value of any particular diet.

